# Ethnic Contrasts in Stroke Risk Factors and the Atrial Fibrillation Paradox in the United Kingdom: Population-Based Study and Meta-Analysis

**DOI:** 10.1212/WNL.0000000000214178

**Published:** 2025-09-23

**Authors:** Joseph Kamtchum-Tatuene, Gabriel S.C. Yiin, Linxin Li, Peter M. Rothwell

**Affiliations:** Wolfson Centre for Prevention of Stroke and Dementia, Nuffield Department of Clinical Neurosciences, University of Oxford, United Kingdom.

## Abstract

**Background and Objectives:**

Studies in northern America report lower prevalence of atrial fibrillation (AF) in Black people than in White people despite higher vascular risk factor prevalence. However, it remains unclear whether these differences are driven by biology vs variations in health care access or alcohol use. We aimed to determine whether ethnic differences in AF persist in the United Kingdom, where the National Health Service provides equitable access to care, and whether they are robust to adjustment for deprivation and alcohol use and are also seen for covert paroxysmal AF on ambulatory screening.

**Methods:**

We performed a systematic review of UK-based studies reporting AF and vascular risk factor prevalence across ethnic groups and pooled estimates by random-effects meta-analysis. Findings were validated in a prospective population-based cohort (Oxford Vascular Study, OxVasc) of patients with suspected TIA or stroke in Oxfordshire, United Kingdom (April 2002–March 2023), through logistic regression adjusted for deprivation and alcohol use, and in a subset of participants recruited after October 2010 who were systematically screened for left atrial dilatation and paroxysmal AF.

**Results:**

Among UK-based studies of patients with stroke, Black and Asian people had lower prevalence of AF (pooled OR, 95% CI, number of studies: 0.25, 0.20–0.32, n = 3; 0.37, 0.28–0.49, n = 6), alcohol consumption (0.42, 0.36–0.49, n = 2; 0.26, 0.13–0.49, n = 3), and smoking (0.70, 0.50–0.97, n = 2; 0.57, 0.44–0.74, n = 5), but higher rates of hypertension (1.95, 1.47–2.60, n = 3; 1.47, 1.02–2.12, n = 6) and diabetes (2.78, 2.40–3.22, n = 3; 4.15, 3.11–5.53, n = 6). In stroke-free populations, similar differences were observed, especially for AF (0.47, 0.12–1.86, n = 2; 0.34, 0.15–0.74, n = 5). Among 7,297 OxVasc participants (47.4% women, 71.0 ± 15.5 years, 335 non-White), AF prevalence was lower in non-White people even after adjustment for age, sex, vascular risk factors, deprivation, and alcohol consumption (adjusted odds ratio [OR] = 0.52, 0.32–0.82, *p* = 0.005). Among 2,221 participants with routine cardiac investigation, non-White people had lower prevalence of paroxysmal AF (2.3% vs 9.1%, OR = 0.24, 0.07–0.75, *p* = 0.004) or atrial dilatation (17.7% vs 27.2%, OR = 0.58, 0.34–0.99, *p* = 0.04).

**Discussion:**

An AF paradox exists in ethnic minority groups in the United Kingdom, for permanent and paroxysmal AF, which is independent of vascular risk factors, deprivation, and alcohol consumption, suggesting different biological susceptibilities.

## Introduction

Atrial fibrillation (AF) accounts for a quarter of the 7.8 million incident ischemic strokes recorded each year globally.^1,2^ Studies in the United States have reported the paradoxical finding that Black and Asian Americans have lower rates of AF than White Americans despite having higher rates of hypertension and other vascular risk factors. For instance, in the US Cardiovascular Health Study, Black Americans had a 45% lower 10-year risk of developing AF than White Americans after adjusting for age, sex, and vascular risk factors.^3^ Similar results have been reported for Black and Asian Americans as well as other ethnic minority groups vs White Americans in several US-based studies.^4-6^ These findings have often been interpreted as indicating a higher biological susceptibility to AF in White Americans, albeit accepting that race and ethnicity are also social constructs. Indeed, the observed differences could at least partly reflect ethnic disparities in health care access in the northern American context.

In the United Kingdom, the publicly funded National Health Service, free at the point of access, provides an opportunity to refute the suggestion that the AF paradox observed in US studies is attributable to access-to-care bias. However, it is also possible that a lower prevalence of permanent AF in Black people and other non-White ethnic groups is offset by a higher prevalence of paroxysmal AF. So far, evidence from US studies does not support this hypothesis, but Black Americans are less likely to receive implantable cardiac monitoring after ischemic stroke.^6^ To our knowledge, no previous UK-based studies have reported on ethnic differences in the prevalence of both permanent and paroxysmal AF in an unselected population. Previous UK studies of ethnic differences in AF prevalence and incidence have relied on medical history or standard electrocardiographic recording^7,8^ and have not adjusted estimates for socioeconomic deprivation or heavier alcohol intake in White people compared with other ethnic groups.^9^ Moreover, some studies, such as the UK Biobank,^8^ have relied on research volunteers, which could introduce bias due to the well-described ethnic differences in likelihood to volunteer for medical research studies.^10^ Ideally, studies of ethnic differences should aim to achieve unbiased inclusion by ensuring that both ascertainment of potential recruits and agreement to participation are as comparable as possible between ethnic groups. One approach is to study patients with stroke, a condition for which most of the people seek medical attention.

We, therefore, aimed to generate unbiased estimates of the magnitude of ethnic differences in AF prevalence across the United Kingdom through a multistep approach. First, we conducted a meta-analysis of published aggregate data. Second, we validated the results in the prospective population-based Oxford Vascular Study (OxVasc)^11^ to allow adjustment for confounders and minimize biases due to selective or incomplete reporting of risk factors. Third, we explored the potential contribution of differences in alcohol consumption in OxVasc to the observed cross-ethnic differences in AF prevalence. Fourth, we assessed whether these differences also exist for covert paroxysmal AF detected on continuous ambulatory cardiac monitoring in OxVasc. Finally, we determined whether similar ethnic differences are observed with left atrial dilatation, a marker of atrial cardiopathy that predicts AF detection.^12^

## Methods

### Search Strategy and Selection Criteria

We searched PubMed and Ovid-EMBASE for studies of ethnic differences in AF and associated stroke risk factors, conducted in the United Kingdom, and published before December 2024. We used an abbreviated search strategy combining “race/ethnicity,” “stroke,” and “United Kingdom” to maximize sensitivity (eTable 1). We incorporated both “ethnicity” and “race” to ensure that we retrieve all publications with potentially relevant information. However, in this report, we follow the latest recommendations of the UK Government Office for Equality and Opportunity to only refer to ethnicity and not race when talking about people who are not from a White British background. Race identifies individuals based solely on their externally observable physical characteristics while ethnicity is a broader self-reported characteristic reflecting various aspects of a person's life including place of birth, nationality, language, skin color, religion, and ancestry.^13^

We excluded studies (1) comparing 1 UK-based ethnic group with a population outside the United Kingdom, (2) describing ethnic differences in AF and vascular risk factors but not in relationship with the risk of stroke, and (3) not enrolling at least 30 participants for each ethnic group. Methodological quality and risk of bias were assessed using an adapted version of the Risk of Bias Tool for Prevalence Studies (eTable 2).^14^ We aimed to exclude studies with high risk of bias. This report follows the Meta-Analysis of Observational Studies guidelines.^15^

### Population-Based Validation Study

Detailed methods of OxVasc have been published previously.^11^ Launched in April 2002, OxVasc enrolls and follows all patients with suspected acute cerebrovascular events from a population of nearly 100,000 individuals registered with approximately 100 primary care physicians across 8 general practices in Oxfordshire, United Kingdom.

Multiple overlapping methods are used to achieve near-complete ascertainment of all patients with suspected vascular events in the study population as reported previously.^11,16^ In addition to prospective daily searches for all hospitalized patients with stroke and regular reviews of multiple administrative sources, the study provides a dedicated daily clinical service, to which the participating general practices refer all patients with suspected TIA or minor stroke for assessment and investigation. Provision of the clinical service facilitates reliable ascertainment of eligible participants, standardized investigation, and consistent diagnosis, with all patients and investigations reviewed by the same Clinical Director since 2002.

All participants undergo a comprehensive assessment including face-to-face interviews, review of hospital and primary care records, brain and vascular imaging (CT, MRI, carotid-vertebral ultrasound), 12-lead electrocardiogram, echocardiography, and routine blood tests. Demographics (age, sex, ethnicity, index of multiple deprivation), stroke risk factors (hypertension, diabetes, smoking, hyperlipidemia, alcohol consumption, and AF), and history of myocardial infarction were recorded. Ethnicity was coded in 4 categories representing Asian people (Bangladeshi, Chinese, Indian, Pakistani), Black people (Black African or Black Caribbean), White people, and other participants (including people from mixed background). The underlying population of OxVasc is 94% White, 4.6% Asian, and 1.4% Black. This matches the composition of the UK-wide population in the decade when OxVasc was initiated (90% White people—2001 UK Census data) and that of the populations of comparable Western countries such as France, Germany, and Australia.^11,17^ Index of multiple deprivation is an overall measure of socioeconomic deprivation obtained through a weighted combination of 7 domain-specific deprivation scores related to income (22.5%), employment (22.5%), education (13.5%), health (13.5%), crime (9.3%), housing (9.3%), and living environment (9.3%).^18^ An index is computed by the Ministry of Housing, Communities and Local Government and the Office of National Statistics for each of 32,844 small geographical areas of England with a similar population size of approximately 1,500 residents or 650 households.^18^ Therefore, it provides a robust, standardized, ready-to-use quantitative measure of the social determinants of health at the individual and community level. Indeed, the 7 domains of the UK index of multiple deprivation overlap almost perfectly with the 5 domains of the social determinants of health as defined by the United States Office of Disease Prevention and Health Promotion,^19^ although there might be minor differences. For instance, the index of multiple deprivation may not fully capture elements of human relationships at home. For OxVasc participants, indices of multiple deprivation were obtained by linking their individual postcodes to the corresponding reference geographical area.

From October 2010 onward, all participants with TIA or minor stroke and without AF (medical history or baseline ECG), either managed initially in the OxVasc clinic service or followed after hospital admission, underwent a 5-day event loop recording of their heart rhythm (R Test Evolution 4, Novacor UK Limited, Lenham, United Kingdom) within approximately 1 month of their index event. The procedure for performing and interpreting the loop recording is described in the eMethods. Echocardiography was also performed within approximately 1 month of the index event to assess left atrial dilatation. Incident AF was identified during long-term face-to-face follow-up and by review of all hospital admissions and new diagnoses in primary care.

### Statistical Analysis

For the meta-analysis, aggregated data were extracted using a predesigned form (list of variables in the eMethods). In studies reporting numeric variables of interest as median with interquartile range, the mean and standard deviation were derived using validated methods.^20^ To compute UK-wide prevalence of AF and associated stroke risk factors in each ethnic group, we pooled study-specific estimates across all studies including participants from the said ethnic group using meta-analysis with random-effects models, after stabilizing the variance with the Freeman-Tuckey double arcsine transformation.^21,22^ However, for direct cross-ethnic comparisons, we restricted the meta-analysis to studies enrolling participants from the 2 ethnic groups of interest (i.e., Black vs White people, Asian vs White people, and Black vs Asian people) to minimize heterogeneity and bias due to cross-study differences in diagnostic criteria. Results are presented as pooled odds ratio (OR) with 95% CI unless stated otherwise. Case-control studies and studies focusing on specific groups (e.g., United Kingdom Prospective Diabetes Study) were not included in meta-analyses. Publication bias was assessed by inspecting funnel plots and performing the Egger test.^23^ Heterogeneity between studies (or subgroups of studies) was assessed with the χ^2^ test on the Cochran *Q* statistic, and the *I*^2^ index was used to quantify the proportion of total variability due to between-study (or subgroups) heterogeneity.^24^

Subgroups analyses were performed to identify potential drivers of the observed ethnic differences in the prevalence of AF and associated stroke risk factors. Definition of subgroups was guided by levels of the factor variable for categorical parameters (e.g., study population and data collection process) or dichotomization at the median across eligible studies for numeric parameters (e.g., year of publication). Comparison of summary statistics between subgroups was performed using a univariable meta-regression with random-effects models as previously described.^25^

To verify the results of our meta-analysis, we performed 3 validation analyses using OxVasc data collected from inception up to March 31, 2023. First, we performed univariable comparisons of the prevalence of AF and associated stroke risk factors across ethnic groups (Asian, Black, and White people) using the χ^2^ test. Second, we fitted multivariable binary logistic regression models to assess the association of ethnicity with AF while adjusting for sex and age (treated as continuous) and then for all other concurrent stroke risk factors, including index of multiple socioeconomic deprivation and excessive alcohol use. This was necessary given previous knowledge that people from ethnic minority groups are typically younger than White people at stroke onset,^26^ that there is a strong association of age and socioeconomic deprivation with AF,^27^ and that excessive alcohol use may contribute to AF pathobiology.^28^ Third, after restricting the data set to participants undergoing systematic R-test screening, we performed a univariable comparison of AF prevalence between White and non-White people (Asian, Black, and other people pooled together because of small numbers) using the Fisher exact test. This was important to test whether ethnic differences in AF prevalence observed in the entire OxVasc data set could be driven by residual ethnic disparities in reporting AF diagnosis or offering AF screening after stroke as described in previous US-based studies.^6^ Fourth, we performed a univariable comparison of the prevalence of left atrial dilatation in White vs non-White people using the Fisher exact test. This was important to verify the epidemiologic coherence of our data, given that left atrial dilatation is a major component of the pathobiological process leading to AF.^29^

In sensitivity analyses, all univariable and multivariable analyses were repeated after (1) pooling Asian, Black, and other people into the same group (non-White people) vs White people; (2) restricting the data set to participants without history of excessive alcohol use at the time of enrollment in OxVasc; and (3) stratifying the analysis by stroke severity (minor stroke or TIA vs major stroke defined as NIH Stroke Scale score ≥5). Finally, we repeated the meta-analysis while including aggregated OxVasc data to provide an up-to-date estimate of the magnitude of cross-ethnic differences in the prevalence of AF and associated stroke risk factors in the United Kingdom when considering all available evidence.

All statistical tests were unpaired and 2-sided, with a significance threshold of *p* ≤ 0.05. The meta-analysis was performed using Stata, version 18 (StataCorp LLC, College Station, TX), and the independent validation analyses were performed using SPSS, version 29 (IBM Corp., Armonk, NY), and Stata, version 15 (StataCorp LLC).

### Standard Protocol Approvals, Registrations, and Patient Consents

OxVasc was approved by the Oxfordshire Research Ethics Committee (OREC A: 05/Q1604/70). For all participants, we obtained written informed consent or assent from their next of kin.

### Data Availability

Aggregate data collated for the meta-analysis can be retrieved from the included source articles or requested from the corresponding study investigators. For reproducibility purposes, the database generated for the meta-analysis and the file containing all the commands used for data curation and analysis will be shared on request to the corresponding author, after all derived articles have been published.

All requests to access OxVasc data should be directed to Professor Peter Rothwell (peter.rothwell@ndcn.ox.ac.uk).

## Results

The database search retrieved 798 reports, of which 76 met the inclusion criteria and were related to 29 individual studies (eFigure 1 and eTable 3). Overall, 40 reports (52.6%) focused on participants with stroke and 32 (42.1%) on participants without stroke. The general characteristics of the included reports are summarized in eTable 4. All studies included in the meta-analysis of AF prevalence were of moderate to high quality (eTable 5).

**Figure 1:**
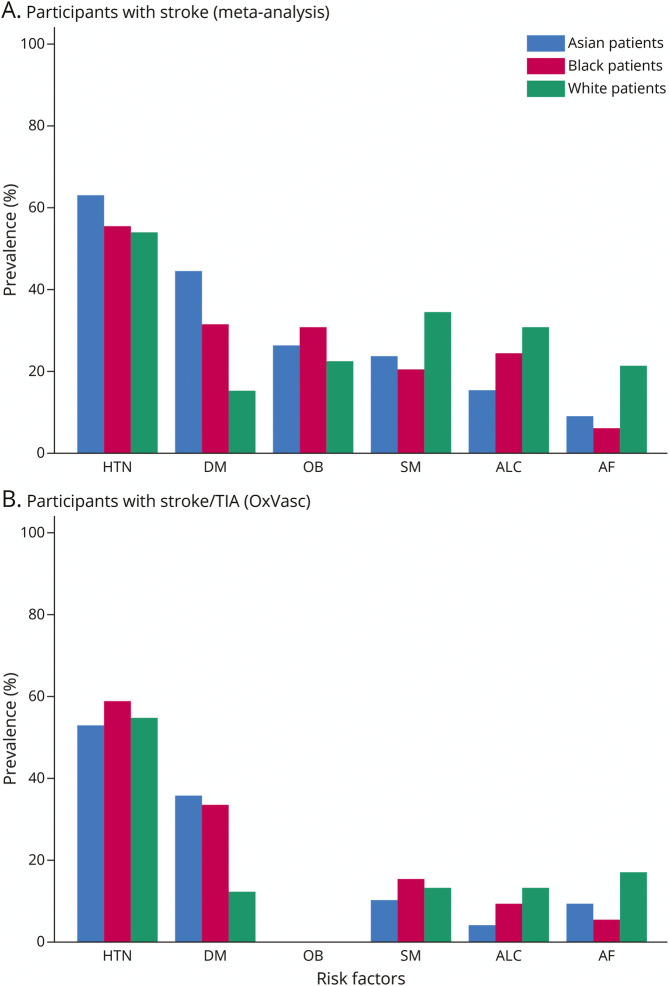
Unadjusted Prevalence Rates Pooled Across Studies of Atrial Fibrillation and Related Risk Factors Stratified by Ethnicity in Previous Studies of Patients With Stroke/TIA and in the Oxford Vascular Study AF = atrial fibrillation; ALC = alcohol consumption; DM = diabetes mellitus; HTN = hypertension; OB = obesity; OxVasc = Oxford Vascular Study; SM = smoking.

Pooled crude prevalence rates of vascular risk factors in people with or without stroke are presented for each ethnic group in Figure 1 and in the Appendix (eTable 6, eFigures 2–38). AF was more prevalent in White people (13.1%, 8.6–18.5, n = 11 studies) than in Asian (4.8%, 2.3–8.2, n = 9) and Black (2.8%, 0.9–5.8, n = 6) people.

**Figure 2:**
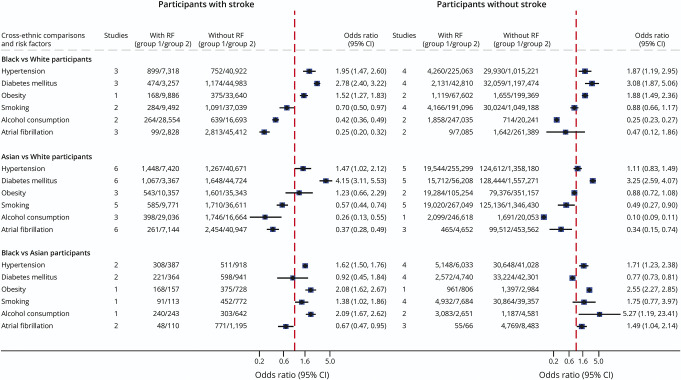
Pooled Estimates of Ethnic Differences in the Unadjusted Prevalence of Atrial Fibrillation and Related Risk Factors Stratified According to Whether the Study Was Performed in Patients With Stroke/TIA or Nonstroke Cohorts Each blue diamond represents the pooled odds ratio for each cross-ethnic comparison of the prevalence of a given risk factor, and the horizontal line on each side of the diamond represents the 95% CI. The vertical dashed red line represents the line of no difference (odds ratio = 1) between the ethnic groups compared.

In the subset of studies performing unadjusted head-to-head cross-ethnic comparisons, the prevalence of AF was lower in Black (pooled OR = 0.26, 95% CI: 0.20–0.33, n = 5) and Asian (0.38, 0.30–0.48, n = 9) people vs White people but no difference was observed between Black and Asian people (1.01, 0.62–1.64, n = 5) (Figure 2, eTables 7–9, eFigures 24–26). However, when restricting analyses to studies enrolling patients without stroke, Black people had a 50% higher AF prevalence than Asian people (1.49, 1.04–2.14, n = 3). The reverse was observed in patients with stroke, with Asian people having a 33% higher AF prevalence than Black people (Figure 2, eTable 9, eFigure 26).

In 4 cohorts reporting on alcohol consumption in Black vs White people, the prevalence was lower in Black people both in studies focusing on participants with stroke (0.42, 0.36–0.49, n = 2)^30,31^ and in those enrolling only people without stroke (0.25, 0.23–0.27, n = 2) (Figure 2, eFigure 36).^32,33^ In 3 hospital-based studies,^30,34,35^ the prevalence of alcohol consumption in patients with stroke was lower in Asians (15.4%, 7.3–25.9) vs White people (37.4%, 13.4–65.4) (Figure 2, eFigures 34, 35, and 37). A similar trend was reported in people without stroke enrolled in the UK Biobank (55.4% vs 92.5%, eFigures 34, 35, and 37).^32^ The prevalence of alcohol consumption was higher in Black vs Asian people in 1 study of patients with stroke (44.2%, 40.1–48.4 vs 27.5%, 24.6–30.5)^30^ and in 2 studies enrolling people without stroke (72.3%, 70.9–73.6 vs 35.3%, 34.2–36.4) (Figure 2, eFigures 33, 35, and 38).^32,36^

A total of 7,297 participants (47.4% women) with suspected acute cerebrovascular events were enrolled in OxVasc up to March 31, 2023, including 6,962 White people (95.4%, 71.0 ± 15.5 years, 47.5% women) who were significantly older than Asian (n = 189, 61.0 ± 16.9, 47.6% women) and Black (n = 117, 62.9 ± 18.0, 45.3% women) people (eTable 10). The validation analysis confirmed the lower prevalence of AF in Black vs White people (adjusted OR = 0.48, 0.24–0.95, *p* = 0.04) after adjustment for age, sex, socioeconomic deprivation, and other vascular risk factors (Table 1). Similar results were obtained after further adjusting for excessive alcohol use (Table 1), pooling all non-White participants into the same group (Tables 1 and 2), restricting the analysis to participants without history of excessive alcohol use at the time of enrollment (Table 2), and stratifying the fully adjusted analyses by stroke severity (adjusted OR = 0.44, 0.25–0.79, *p* = 0.005 for minor stroke or TIA; adjusted OR = 0.54, 0.20–1.44, *p* = 0.22, for major stroke).

**Table 1:** Direct Cross-Ethnic Comparisons of Prevalence of Atrial Fibrillation and Related Risk Factors in Patients With Suspected TIA or Stroke in the Oxford Vascular Study

	OR1 (95% CI)^a^	*p* Value	OR2 (95% CI)^b^	*p* Value	OR3 (95% CI)^c^	*p* Value	OR4 (95% CI)^d^	*p* Value
Non-White vs White people								
History of atrial fibrillation	0.39 (0.25–0.61)	<0.001	0.65 (0.41–1.02)	0.06	0.61 (0.38–0.97)	0.04	0.54 (0.31–0.94)	0.03
Any atrial fibrillation^e^	0.40 (0.27–0.57)	<0.001	0.64 (0.43–0.94)	0.02	0.59 (0.40–0.88)	0.04	0.52 (0.32–0.82)	0.005
Hypertension	0.94 (0.76–1.17)	0.59	1.46 (1.16–1.85)	0.002	1.13 (0.87–1.47)	0.36	1.13 (0.84–1.51)	0.43
Diabetes mellitus	3.26 (2.55–4.16)	<0.001	3.96 (3.07–5.10)	<0.001	3.33 (2.52–4.39)	<0.001	2.94 (2.15–4.02)	<0.001
Hyperlipidemia	1.13 (0.89–1.43)	0.34	1.32 (1.03–1.69)	0.03	1.08 (0.82–1.42)	0.57	1.16 (0.86–1.56)	0.34
Current smoking	0.92 (0.66–1.29)	0.63	0.58 (0.40–0.82)	0.002	0.46 (0.32–0.66)	<0.001	0.51 (0.34–0.76)	<0.001
Previous myocardial infarction	0.73 (0.46–1.16)	0.19	1.03 (0.64–1.65)	0.90	0.90 (0.55–1.47)	0.66	0.79 (0.46–1.38)	0.41
Previous peripheral vascular disease	0.44 (0.21–0.93)	0.03	0.61 (0.28–1.28)	0.18	0.41 (0.19–0.91)	0.03	0.30 (0.11–0.83)	0.02
Alcohol intake >14 units per week	0.39 (0.24–0.63)	<0.001	0.31 (0.19–0.50)	<0.001	0.35 (0.21–0.58)	<0.001	—	—
Black vs White people								
History of atrial fibrillation	0.25 (0.10–0.61)	0.003	0.37 (0.15–0.91)	0.03	0.34 (0.140.87)	0.02	0.45 (0.18–1.16)	0.10
Any atrial fibrillation^e^	0.35 (0.18–0.67)	0.002	0.50 (0.26–0.99)	0.046	0.48 (0.24–0.95)	0.04	0.47 (0.22–1.00)	0.049
Hypertension	1.09 (0.76–1.58)	0.64	1.59 (1.07–2.36)	0.02	1.35 (0.87–2.07)	0.18	1.40 (0.87–2.26)	0.17
Diabetes mellitus	3.23 (2.16–4.82)	<0.001	3.80 (2.52–5.72)	<0.001	3.16 (2.03–4.92)	<0.001	2.88 (1.76–4.71)	<0.001
Hyperlipidemia	0.78 (0.50–1.21)	0.26	0.89 (0.57–1.38)	0.59	0.72 (0.45–1.15)	0.17	0.78 (0.47–1.29)	0.33
Current smoking	1.14 (0.68–1.92)	0.62	0.77 (0.45–1.34)	0.36	0.58 (0.33–1.00)	0.05	0.58 (0.31–1.07)	0.08
Previous myocardial infarction	0.31 (0.10–0.98)	0.046	0.55 (0.20–1.52)	0.25	0.50 (0.18–1.41)	0.19	0.53 (0.18–1.50)	0.23
Previous peripheral vascular disease	0.36 (0.09–1.45)	0.15	0.48 (0.12–1.96)	0.31	0.34 (0.08–1.46)	0.15	0.19 (0.03–1.44)	0.11
Alcohol intake >14 units per week	0.62 (0.32–1.20)	0.16	0.52 (0.26–1.02)	0.06	0.58 (0.29–1.16)	0.12	—	—
Asian vs White people								
History of atrial fibrillation	0.45 (0.26–0.78)	0.004	0.76 (0.43–1.35)	0.35	0.71 (0.40–1.26)	0.24	0.65 (0.33–1.29)	0.22
Any atrial fibrillation^e^	0.40 (0.24–0.64)	<0.001	0.65 (0.39–1.08)	0.10	0.59 (0.35–1.00)	0.05	0.56 (0.30–1.01)	0.06
Hypertension	0.87 (0.65–1.16)	0.34	1.35 (0.99–1.84)	0.06	0.95 (0.67–1.34)	0.76	0.97 (0.66–1.43)	0.89
Diabetes mellitus	3.78 (2.77–5.16)	<0.001	4.59 (3.34–6.32)	<0.001	3.93 (2.77–5.57)	<0.001	3.29 (2.22–4.88)	<0.001
Hyperlipidemia	1.43 (1.05–1.94)	0.02	1.68 (1.23–2.29)	0.001	1.37 (0.97–1.94)	0.07	1.43 (0.98–2.08)	0.07
Current smoking	0.68 (0.41–1.12)	0.13	0.42 (0.25–0.71)	0.001	0.35 (0.20–0.59)	<0.001	0.41 (0.23–0.73)	<0.001
Previous myocardial infarction	1.01 (0.59–1.72)	0.98	1.40 (0.81–2.42)	0.23	1.18 (0.67–2.10)	0.57	0.95 (0.49–1.84)	0.88
Previous peripheral vascular disease	0.56 (0.23–1.36)	0.20	0.75 (0.30–1.85)	0.53	0.51 (0.20–1.30)	0.16	0.40 (0.12–1.30)	0.13
Alcohol intake >14 units per week	0.22 (0.10–0.50)	<0.001	0.18 (0.08–0.40)	<0.001	0.21 (0.09–0.48)	<0.001	—	—

a OR1 corresponds to the unadjusted odds ratio (univariable comparison).

b OR2 corresponds to the odds ratio obtained using a multivariable binary logistic regression adjusted for sex and age (treated as continuous).

c OR3 corresponds to the odds ratio obtained using a multivariable binary logistic regression adjusted for sex, age (treated as continuous), index of multiple deprivation (treated as continuous), and all other vascular risk factors. Any atrial fibrillation was included as a covariate in the adjusted model for all risk factors except for history of atrial fibrillation.

d OR4 corresponds to the odds ratio obtained using a multivariable binary logistic regression adjusted for sex, age (treated as continuous), index of multiple deprivation (treated as continuous), and all other vascular risk factors including alcohol use >14 units per week. Any atrial fibrillation was included as a covariate in the adjusted model for all risk factors except for history of atrial fibrillation. Only those with known alcohol status were included (n = 5,598).

e Any atrial fibrillation includes history of atrial fibrillation and any episode of atrial fibrillation detected after the index event (admission ECG, in-hospital telemetry, or ambulatory R-test).

**Table 2:** Direct Comparison of Prevalence of Alcohol Use, AF, and Left Atrial Dilatation in Non-White vs White Oxford Vascular Study Participants

	Non-White people, n (%)	White people, n (%)	Crude OR (95% CI)	*p* Value	Adjusted OR (95% CI)^a^	*p* Value
All participants from inception (2002–2023; n = 7,297)						
All events						
Alcohol intake >14 units per week^b^	18 (6.9)	860 (16.1)	0.39 (0.24–0.63)	<0.001	—	—
History of AF	22 (6.6)	1,053 (15.1)	0.39 (0.25–0.61)	<0.001	0.54 (0.31–0.94)	0.03
Any AF	32 (9.6)	1,464 (21.0)	0.40 (0.27–0.57)	<0.001	0.52 (0.32–0.82)	0.005
Excluding history of excessive alcohol use						
History of AF	21 (6.3)	1,037 (15.2)	0.38 (0.24–0.59)	<0.001	0.51 (0.29–0.90)	0.02
Any AF	31 (9.3)	1,441 (21.1)	0.39 (0.27–0.56)	<0.001	0.50 (0.31–0.81)	0.004
All inpatients and outpatients after implementation of systematic cardiac monitoring in outpatients (2010–2023; n = 4,597)						
All events						
Alcohol intake >14 units per week^b^	11 (5.5)	562 (16.1)	0.31 (0.17–0.56)	<0.001	—	—
History of AF	15 (6.1)	631 (14.5)	0.38 (0.22–0.64)	<0.001	0.60 (0.31–1.15)	0.12
Any AF	21 (8.5)	930 (21.4)	0.34 (0.22–0.54)	<0.001	0.49 (0.28–0.85)	0.01
Excluding history of excessive alcohol use						
History of AF	14 (5.7)	620 (14.6)	0.36 (0.21–0.62)	<0.001	0.56 (0.28–1.10)	0.092
Any AF	20 (8.2)	912 (21.4)	0.33 (0.21–0.52)	<0.001	0.47 (0.27–0.83)	0.009
All events						
Alcohol intake >14 units per week^b^	6 (4.7)	337 (16.7)	0.25 (0.11–0.56)	<0.001	—	—
Any AF on R-test	3 (2.3)	190 (9.1)	0.23 (0.07–0.74)	0.004	0.29 (0.09–0.96)	0.04
Any AF >30s on R-test	0 (0)	92 (4.4)	—	0.006	—	—
No left atrial dilatation	79 (84.9)	1,081 (73.0)	1.00	0.02^c^	1.00	—
Mild left atrial dilatation	10 (10.8)	270 (18.2)	0.51 (0.26–0.99)		0.58 (0.29–1.18)	0.13
Moderate/severe left atrial dilatation	4 (4.3)	129 (8.7)	0.42 (0.15–1.18)		0.70 (0.24–2.03)^d^	0.51
Any left atrial dilatation	17 (17.7)	403 (27.2)	0.58 (0.34–0.99)	0.04	0.74 (0.41–1.31)	0.29
Excluding history of excessive alcohol use						
Any AF on R-test	3 (2.3)	186 (9.1)	0.24 (0.07–0.75)	0.004	0.30 (0.09–0.97)	0.045
Any AF >30s on R-test	0 (0)	91 (4.4)	—	0.006	—	—
No left atrial dilatation	79 (84.9)	1,061 (73.0)	1.00	0.02^c^	1.00	—
Mild left atrial dilatation	10 (10.8)	264 (18.2)	0.51 (0.26–1.00)		0.58 (0.28–1.17)	0.13
Moderate/severe left atrial dilatation	4 (4.3)	128 (8.8)	0.42 (0.15–1.17)		0.70 (0.24–2.02)^d^	0.51
Any left atrial dilatation	17 (17.7)	396 (27.2)	0.58 (0.34–0.99)	0.04	0.73 (0.41–1.30)	0.29

Abbreviations: AF = atrial fibrillation; OR = odds ratio.

a Adjusted for sex, age (treated as continuous), alcohol use >14 units per week, history of hypertension, diabetes, hyperlipidemia, current smoking, previous myocardial infarction, previous peripheral vascular disease, and index of multiple deprivation (treated as continuous).

b Based only on patients with alcohol intake ascertained. Whole cohort from inception: n = 5,598 for all events and n = 5,474 after excluding history of excessive alcohol use. Participants after implementation of systematic cardiac monitoring: n = 3,690 for all events and n = 3,604 after excluding history of excessive alcohol use. Participants effectively undergoing the R-test: n = 2,148 for all events and n = 2,112 excluding history of excessive alcohol use.

c *p* Value for a trend test across all categories of left atrial dilatation. Left atrium size was measured on transthoracic echocardiography for 1,580 participants. Left atrial dilatation was diagnosed in 420, including 17 of 96 non-Whites (17.7%) and 403 of 1,484 Whites (27.2%). Left atrial dilatation was described as moderate to severe in 1 non-White participant, and the severity was not specified in 3 non-Whites and 4 Whites. Therefore, classification of left atrial dilatation severity was only possible for 1,573 participants (93 non-Whites, 1,480 Whites) in the main analysis and 1,546 participants (93 non-Whites, 1,453 Whites) after excluding those with history of excessive alcohol use.

d For the comparison between moderate/severe and no/mild left atrial dilatation.

From October 2010 onward, OxVasc enrolled 4,597 participants (246 non-White) with acute stroke or TIA (Table 2), including 2,221 outpatients (48.6% women, 67.5 ± 14.2 years, 131 non-White) who underwent a 5-day ambulatory cardiac monitoring (eTable 11). Adequate left atrial assessment by echocardiography was obtained in 1,580 (71.1%, 96 non-White). Screening rates were similar in non-Whites vs White people for both cardiac monitoring (53.3% vs 48.03%, *p* = 0.11) and echocardiography (39.0% vs 34.1%, *p* = 0.11). AF was diagnosed in 193 participants and left atrial dilatation in 420 (eTable 11). Non-White people were less likely to have either paroxysmal AF (2.3% vs 9.1%, OR = 0.23, 0.07–0.74, *p* = 0.004) or left atrial dilatation (17.7% vs 27.2%, OR = 0.58, 0.34–0.99, *p* = 0.04) than White people (Table 2). Similar results were obtained in the sensitivity analyses (Table 2). In 1,838 participants without AF on their initial ambulatory cardiac monitoring (1,722 White and 116 non-White people), additional incident AF cases were identified during long-term follow-up in 3 non-White and 105 White people (2.6% vs 6.1%, OR = 0.41, 0.13–1.31, *p* = 0.13).

**Figure 3:**
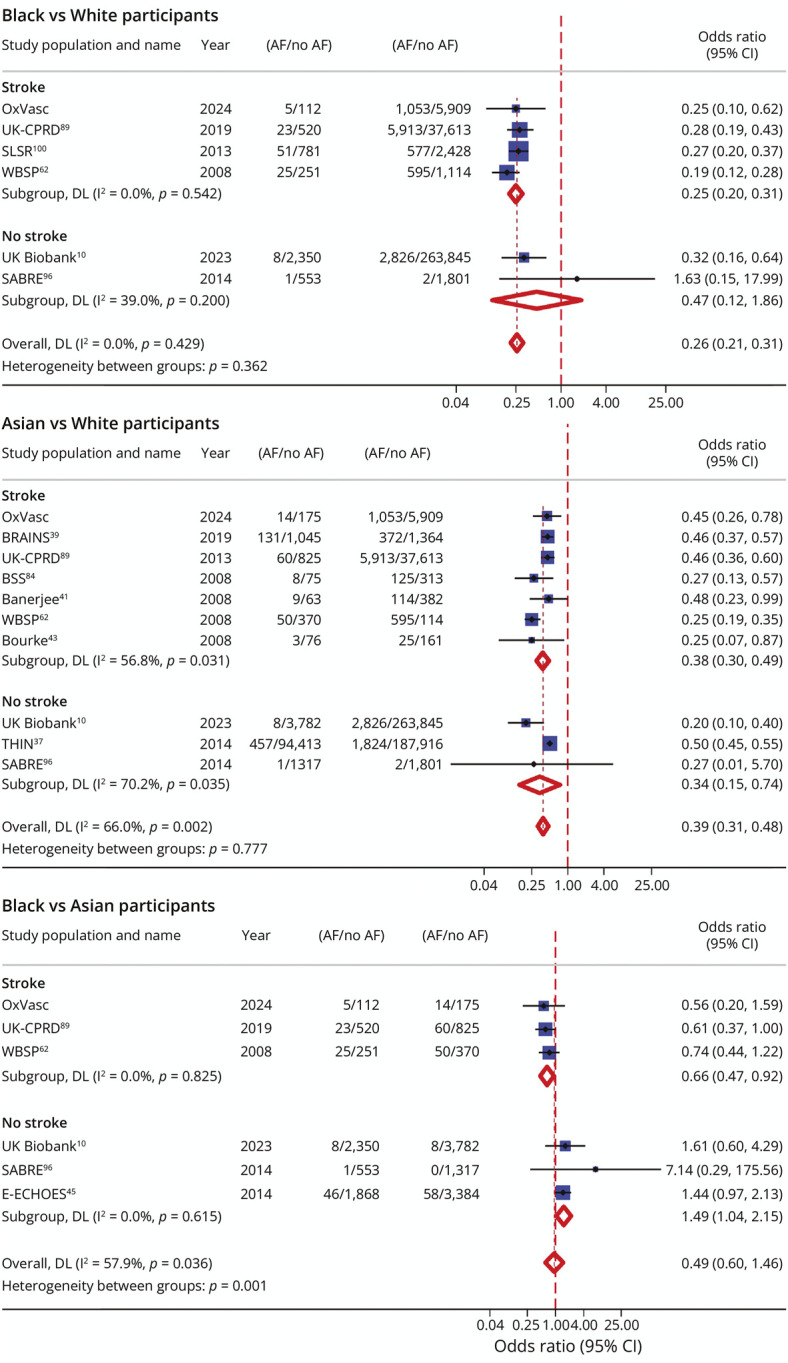
Meta-Analysis of Ethnic Differences in the Unadjusted Prevalence of Atrial Fibrillation Stratified According to Whether the Study Was Performed in Patients With Stroke/TIA or Nonstroke Cohorts AF = atrial fibrillation; BRAINS = Biorepository of DNA in Stroke; BSS = Bradford Stroke Study; DL = DerSimonian and Laird inverse variance weighing; E-ECHOES = Ethnic-Echocardiographic Heart of England Screening; NA = not available; SABRE = Southall and Brent Revisited; SLSR = South London Stroke Register; THIN = The Health Improvement Initiative Network; UK Biobank = United Kingdom Biobank; UK-CPRD = United Kingdom Clinical Practice Research Datalink; WBSP = West Birmingham Stroke Project.

In the updated meta-analysis, the reported cross-ethnic differences in prevalence of AF and related vascular risk factors remained substantial (Figure 3, eFigures 39 and 40).

## Discussion

Our results confirm the existence of an “atrial fibrillation paradox” in the United Kingdom and provide evidence that it is not explained by disparities in access to AF screening as previously described in US studies enrolling stroke-free participants.^37-39^ The paradox exists not only in Black people but also in Asian people living in the United Kingdom. It is independent of the distribution of stroke risk factors, including smoking, alcohol consumption, and socioeconomic deprivation. Indeed, the lower likelihood of AF in ethnic minority groups, especially Black people, persists in multivariable models and in sensitivity analyses restricted to participants with limited alcohol use.

The lower prevalence of AF in ethnic minority groups vs White people has traditionally been attributed to age differences and disparities in access to screening. However, although White people live longer and are 5–10 years older than Asian and Black people at the time of stroke onset, studies of age-stratified AF prevalence report similar ethnic differences in AF burden across all age groups.^40^ In addition, the impact of any disparities in access to screening would likely be smaller in the United Kingdom where there are fewer inequalities in access to care than in the United States.^41^ Moreover, our analyses of data from OxVasc participants undergoing systematic ambulatory cardiac monitoring after suspected TIA or stroke irrespective of ethnicity confirmed the ethnic differences in AF prevalence. The lower prevalence of moderate-to-severe left atrial dilatation in Black and Asian people in OxVasc could also be interpreted as an indirect marker of lower biological susceptibility to atrial cardiopathy, a precursor of AF.^12^

Previous studies of ethnic differences in susceptibility to AF have highlighted genetic influences. While Black people have a higher prevalence of *rs10824026*, a single-nucleotide variations that is protective against AF,^42,43^ they have a higher prevalence of AF-promoting single-nucleotide variations at chromosomes 4q25, 1q21, and 16q22^43^ and a higher prevalence of pathogenic and likely pathogenic variants of other genes promoting early-onset AF, compared with White people.^44^ Although important, these preliminary genetic findings do not fully explain clinical and epidemiologic differences between Asian, Black, and White people regarding the susceptibility to AF and the risk of AF-related adverse cardiovascular outcomes.^39^ Future research in genetically diverse African populations might identify novel protective single-nucleotide variations or genetic variants whose effect was not apparent in previous studies focusing on predominantly White Western populations.^45^

Indeed, our meta-analysis highlights the heterogeneity of findings among ethnic minority groups. For instance, in stroke-free individuals, AF is more prevalent in Black than Asian people while the reverse is true in patients with stroke. There also seems to be an AF paradox when comparing Black with Asian people. Black people have the worse cardiometabolic profile with higher prevalence of hypertension, diabetes, and obesity than White people and higher prevalence of smoking and alcohol consumption than Asian people, irrespective of stroke status. With a few exceptions, and despite small numbers of participants in ethnic minority groups, these observations were verified in OxVasc, thus emphasizing that clinical and genetic information obtained in 1 ethnic minority group are not necessarily applicable to another.^45,46^ In other words, although Black and Asian people seem to be less susceptible to AF than White people, this may be explained by different pathobiological processes, thus highlighting the need for more diversity in future genomic studies of AF.^45^

Our study has several strengths. First, it provides a comprehensive meta-analysis with systematic comparison of stroke risk factor and AF prevalence between Asian, Black, and White people across the United Kingdom. Second, it also reports important observations from a UK-based prospective investigation of cross-ethnic differences in AF prevalence in a cohort with universal access to cardiac monitoring to minimize access-to-care bias. Third, relying on data from a prospective population-based cohort, recruited at the point of seeking medical attention and responsible for providing clinical care, helped ensure that estimates of AF prevalence are not affected by the lower likelihood of ethnic minority groups participating in research.^10^

Nevertheless, there are some limitations. First, ascertainment in OxVasc requires patients to seek medical attention and so some underrepresentation of ethnic minority groups is possible because of their lower willingness to seek medical attention.^26,47-49^ However, the distribution of OxVasc participants by age, sex, and ethnicity has remained consistently similar to that of the underlying population in South East England from inception.^11,17,50^

Second, in our meta-analysis, there were multiple sources of heterogeneity across the included studies, notably the definition of vascular risk factors, the age range and place of residence of participants, and the time of and approach to data collection. Nevertheless, estimates of the cross-ethnic differences in prevalence of AF and its associated risk factors were relatively consistent across studies and subgroups and were comparable with those in OxVasc. Third, the overall number of participants from ethnic minority groups remained relatively small even after combining all available data, and there was limited information on the composition of aggregated ethnic minority groups. This means that some differences between various strata of the population of UK residents who are not originally from a White British background (e.g., Black African vs Black Caribbean people and Asian people from India vs China) might have been overlooked. This also means that we could not perform in-depth exploration of the contribution of other factors such as early-life stressors (e.g., infections, pollution, and malnutrition), migrant generation, and time since immigration that determines the cumulative exposure to western lifestyle and public health interventions. Further studies enrolling larger numbers of people from ethnic minority groups are, therefore, needed to properly address the highlighted gaps in knowledge.

Overall, our findings have 3 implications. First, the lower susceptibility to AF in Black and Asian people suggests that considering ethnicity might improve both the accuracy and the generalizability of scores to predict AF detection after TIA and stroke. Second, future research should aim to quantify the benefit of genetic testing in ethnic minority groups with AF-related cardioembolic strokes, given their higher prevalence of pathogenic variations of genes promoting early-onset AF.^44^ Third, more in-depth investigations of the biological mechanisms underpinning the AF paradox, ideally including comparisons between people of African/Asian descent living in Western countries and those remaining in Africa or Asia, could lead to the discovery of novel therapeutic targets for AF prevention and treatment and might also inform guidelines to meet the specific needs of ethnic minority groups.

## Glossary

AF: atrial fibrillation
OR: odds ratio
OxVasc: Oxford Vascular Study

## References

[1] Elsheikh S, Hill A, Irving G, Lip GYH, Abdul-Rahim AH. Atrial fibrillation and stroke: state-of-the-art and future directions. Curr Probl Cardiol. 2024;49(1 pt C):102181. doi:10.1016/j.cpcardiol.2023.102181 37913929

[2] Global Burden of Diseases Stroke Risk Factor Collaborators. Global, regional, and national burden of stroke and its risk factors, 1990-2021: a systematic analysis for the Global Burden of Disease Study 2021. Lancet Neurol. 2024;23(10):973-1003. doi:10.1016/s1474-4422(24)00369-7 39304265 PMC12254192

[3] Jensen PN, Thacker EL, Dublin S, Psaty BM, Heckbert SR. Racial differences in the incidence of and risk factors for atrial fibrillation in older adults: the cardiovascular health study. J Am Geriatr Soc. 2013;61(2):276-280. doi:10.1111/jgs.12085 23320758 PMC3878638

[4] Heckbert SR, Austin TR, Jensen PN, et al. Differences by race/ethnicity in the prevalence of clinically detected and monitor-detected atrial fibrillation: MESA. Circ Arrhythm Electrophysiol. 2020;13(1):e007698. doi:10.1161/CIRCEP.119.007698 31934795 PMC7204495

[5] Lau CP, Gbadebo TD, Connolly SJ, et al. Ethnic differences in atrial fibrillation identified using implanted cardiac devices. J Cardiovasc Electrophysiol. 2013;24(4):381-387. doi:10.1111/jce.12066 23356818

[6] Yaghi S, Furie K, Wechsler L, et al. Differences in inpatient insertable cardiac monitor placement after ischemic stroke. J Stroke Cerebrovasc Dis. 2022;31(1):106124. doi:10.1016/j.jstrokecerebrovasdis.2021.106124 34674901

[7] Markus HS, Khan U, Birns J, et al. Differences in stroke subtypes between black and white patients with stroke: the South London Ethnicity and Stroke Study. Circulation. 2007;116(19):2157-2164. doi:10.1161/CIRCULATIONAHA.107.699785 17967776

[8] Frimodt-Moller EK, Tang JJ, Biering-Sorensen T, Delling FN, Jackson LR II, Marcus GM. Ethnic differences in atrial fibrillation in the United Kingdom. JACC Adv. 2024;3(12):101043. doi:10.1016/j.jacadv.2024.101043 39817084 PMC11734043

[9] Bhatti SN, Fan LM, Collins A, Li JM. Exploration of alcohol consumption behaviours and health-related influencing factors of young adults in the UK. Int J Environ Res Public Health. 2020;17:6282. doi:10.3390/ijerph17176282 32872341 PMC7503755

[10] Smart A, Harrison E. The under-representation of minority ethnic groups in UK medical research. Ethn Health. 2017;22(1):65-82. doi:10.1080/13557858.2016.1182126 27174778

[11] Rothwell PM, Coull AJ, Giles MF, et al. Change in stroke incidence, mortality, case-fatality, severity, and risk factors in Oxfordshire, UK from 1981 to 2004 (Oxford Vascular Study). Lancet. 2004;363(9425):1925-1933. doi:10.1016/S0140-6736(04)16405-2 15194251

[12] Lim DJ, Ambale-Ventakesh B, Ostovaneh MR, et al. Change in left atrial function predicts incident atrial fibrillation: the Multi-Ethnic Study of Atherosclerosis. Eur Heart J Cardiovasc Imaging. 2019;20(9):979-987. doi:10.1093/ehjci/jez176 31356656 PMC6704390

[13] Office for Equality and Opportunity. Standards for ethnicity data. 2023. Accessed June 12, 2025. gov.uk/government/publications/standards-for-ethnicity-data/standards-for-ethnicity-data.

[14] Hoy D, Brooks P, Woolf A, et al. Assessing risk of bias in prevalence studies: modification of an existing tool and evidence of interrater agreement. J Clin Epidemiol. 2012;65:934-939. doi:10.1016/j.jclinepi.2011.11.014 22742910

[15] Stroup DF, Berlin JA, Morton SC, et al. Meta-analysis of observational studies in epidemiology: a proposal for reporting. Meta-analysis Of Observational Studies in Epidemiology (MOOSE) group. JAMA. 2000;283(15):2008-2012. doi:10.1001/jama.283.15.2008 10789670

[16] Howard DPJ, Gaziano L, Rothwell PM; Oxford Vascular Study. Risk of stroke in relation to degree of asymptomatic carotid stenosis: a population-based cohort study, systematic review, and meta-analysis. Lancet Neurol. 2021;20(3):193-202. doi:10.1016/S1474-4422(20)30484-1 33609477 PMC7889579

[17] Office for National Statistics. UK 2001 Census. 2001. Accessed June 9, 2024. nomisweb.co.uk/sources/census_2001.

[18] Ministry of Housing Communities and Local Government. English indices of deprivation 2019. 2019. Accessed June 13, 2025. gov.uk/government/statistics/english-indices-of-deprivation-2019.

[19] U.S. Department of Health and Human Services - Office of Disease Prevention and Health Promotion. Healthy People 2030: Social Determinants of Health. 2020. Accessed July 8, 2025. odphp.health.gov/healthypeople/priority-areas/social-determinants-health.

[20] McGrath S, Zhao X, Steele R, Thombs BD, Benedetti A; DEPRESsion Screening Data DEPRESSD Collaboration. Estimating the sample mean and standard deviation from commonly reported quantiles in meta-analysis. Stat Methods Med Res. 2020;29(9):2520-2537. doi:10.1177/0962280219889080 32292115 PMC7390706

[21] Freeman MF, Tukey JW. Transformations related to the angular and the square root. Ann Math Statist. 1950;21(4):607-611. doi:10.1214/aoms/1177729756

[22] Nyaga VN, Arbyn M, Aerts M. Metaprop: a Stata command to perform meta-analysis of binomial data. Arch Public Health. 2014;72(1):39. doi:10.1186/2049-3258-72-39 25810908 PMC4373114

[23] Sterne JA, Egger M, Moher D. Addressing reporting biases. In: Higgins JP, Green S, editors. Cochrane Handbook for Systematic Reviews of Interventions. Wiley; 2008:297-334.

[24] Huedo-Medina TB, Sánchez-Meca J, Marín-Martínez F, Botella J. Assessing heterogeneity in meta-analysis: Q statistic or I^2^ index? Psychol Methods. 2006;11(2):193-206. doi:10.1037/1082-989X.11.2.193 16784338

[25] Kamtchum-Tatuene J, Noubiap JJ, Wilman AH, Saqqur M, Shuaib A, Jickling GC. Prevalence of high-risk plaques and risk of stroke in patients with asymptomatic carotid stenosis: a meta-analysis. JAMA Neurol. 2020;77(12):1524-1535. doi:10.1001/jamaneurol.2020.2658 32744595 PMC7400201

[26] Gulli G, Rutten-Jacobs LC, Kalra L, Rudd AG, Wolfe CDA, Markus HS. Differences in the distribution of stroke subtypes in a UK black stroke population: final results from the South London Ethnicity and Stroke Study. BMC Med. 2016;14:77. doi:10.1186/s12916-016-0618-2 27197724 PMC4873985

[27] Chung SC, Sofat R, Acosta-Mena D, et al. Atrial fibrillation epidemiology, disparity and healthcare contacts: a population-wide study of 5.6 million individuals. Lancet Reg Health Eur. 2021;7:100157. doi:10.1016/j.lanepe.2021.100157 34405204 PMC8351189

[28] Brundel B, Ai X, Hills MT, Kuipers MF, Lip GYH, de Groot NMS. Atrial fibrillation. Nat Rev Dis Primers. 2022;8(1):21. doi:10.1038/s41572-022-00347-9 35393446

[29] Dewland TA, Bibbins-Domingo K, Lin F, et al. Racial differences in left atrial size: results from the coronary artery risk development in young adults (CARDIA) study. PLoS One. 2016;11(3):e0151559. doi:10.1371/journal.pone.0151559 26985672 PMC4795666

[30] Shiekh SI, Forbes H, Mathur R, Smeeth L, Pearce N, Warren-Gash C. Ethnicity and risk of diagnosed dementia after stroke: a cohort study using the Clinical Practice Research Datalink. J Epidemiol Community Health. 2020;74(2):114-119. doi:10.1136/jech-2019-212825 31699799 PMC6993022

[31] Wolfe CD, Smeeton NC, Coshall C, Tilling K, Rudd AG. Survival differences after stroke in a multiethnic population: follow-up study with the South London stroke register. BMJ. 2005;331(7514):431. doi:10.1136/bmj.38510.458218.8F 16055452 PMC1188108

[32] Bonnechère B, Liu J, Thompson A, Amin N, van Duijn C. Does ethnicity influence dementia, stroke and mortality risk? Evidence from the UK Biobank. Front Public Health. 2023;11:1111321. doi:10.3389/fpubh.2023.1111321 37124771 PMC10140594

[33] Shibata D, Tillin T, Beauchamp N, et al. African Caribbeans have greater subclinical cerebrovascular disease than Europeans: this is associated with both their elevated resting and ambulatory blood pressure and their hyperglycaemia. J Hypertens. 2013;31(12):2391-2399. doi:10.1097/HJH.0b013e328364f5bc 24029870 PMC4082237

[34] Aurelius T, Maheshwari A, Ken-Dror G, et al. Ischaemic stroke in South Asians: the BRAINS study. Eur J Neurol. 2023;30(2):353-361. doi:10.1111/ene.15605 36260058 PMC10098949

[35] Ramadan H, Patterson C, Maguire S, et al. Incidence of first stroke and ethnic differences in stroke pattern in Bradford, UK: Bradford Stroke Study. Int J Stroke. 2018;13(4):374-378. doi:10.1177/1747493017743052 29192873

[36] Calvert M, Duffy H, Freemantle N, Davis R, Lip GYH, Gill P. Population health status of South Asian and African-Caribbean communities in the United Kingdom. BMC Health Serv Res. 2012;12:101. doi:10.1186/1472-6963-12-101 22533538 PMC3349523

[37] Marcus GM, Alonso A, Peralta CA, et al. European ancestry as a risk factor for atrial fibrillation in African Americans. Circulation. 2010;122(20):2009-2015. doi:10.1161/CIRCULATIONAHA.110.958306 21098467 PMC3058884

[38] Stamos TD, Darbar D. The “double” paradox of atrial fibrillation in black individuals. JAMA Cardiol. 2016;1(4):377-379. doi:10.1001/jamacardio.2016.1259 27438310

[39] Magnani JW, Norby FL, Agarwal SK, et al. Racial differences in atrial fibrillation-related cardiovascular disease and mortality: the Atherosclerosis Risk in Communities (ARIC) study. JAMA Cardiol. 2016;1(4):433-441. doi:10.1001/jamacardio.2016.1025 27438320 PMC5347977

[40] Mathur R, Pollara E, Hull S, Schofield P, Ashworth M, Robson J. Ethnicity and stroke risk in patients with atrial fibrillation. Heart. 2013;99(15):1087-1092. doi:10.1136/heartjnl-2013-303767 23720487

[41] Nazroo JY, Falaschetti E, Pierce M, Primatesta P. Ethnic inequalities in access to and outcomes of healthcare: analysis of the Health Survey for England. J Epidemiol Community Health. 2009;63(12):1022-1027. doi:10.1136/jech.2009.089409 19622520

[42] Darbar D, Roden DM. Genetic mechanisms of atrial fibrillation: impact on response to treatment. Nat Rev Cardiol. 2013;10(6):317-329. doi:10.1038/nrcardio.2013.53 23591267 PMC3664212

[43] Roberts JD, Hu D, Heckbert SR, et al. Genetic investigation into the differential risk of atrial fibrillation among black and white individuals. JAMA Cardiol. 2016;1(4):442-450. doi:10.1001/jamacardio.2016.1185 27438321 PMC5395094

[44] Chalazan B, Mol D, Darbar FA, et al. Association of rare genetic variants and early-onset atrial fibrillation in ethnic minority individuals. JAMA Cardiol. 2021;6(7):811-819. doi:10.1001/jamacardio.2021.0994 33950154 PMC8100900

[45] Fatumo S, Choudhury A. African American genomes don't capture Africa's genetic diversity. Nature. 2023;617(7959):35. doi:10.1038/d41586-023-01479-y 37130934

[46] Kamiza AB, Toure SM, Vujkovic M, et al. Transferability of genetic risk scores in African populations. Nat Med. 2022;28:1163-1166. doi:10.1038/s41591-022-01835-x 35654908 PMC9205766

[47] Fluck D, Fry CH, Gulli G, et al. Adverse stroke outcomes amongst UK ethnic minorities: a multi-centre registry-based cohort study of acute stroke. Neurol Sci. 2023;44(6):2071-2080. doi:10.1007/s10072-023-06640-z 36723729 PMC9891657

[48] Cummings C, Almallouhi E, Al Kasab S, Spiotta AM, Holmstedt CA. Blacks are less likely to present with strokes during the COVID-19 pandemic: observations from the buckle of the stroke belt. Stroke. 2020;51(10):3107-3111. doi:10.1161/STROKEAHA.120.031121 32755454 PMC7434003

[49] Nwokoroku SC, Neil B, Dlamini C, Osuchukwu VC. A systematic review of the role of culture in the mental health service utilisation among ethnic minority groups in the United Kingdom. Glob Ment Health (Camb). 2022;9:84-93. doi:10.1017/gmh.2022.2 36618728 PMC9806997

[50] Office for National Statistics. Ethnic group by age and sex, England and Wales: UK Census 2021. 2021. Accessed December 6, 2024. ons.gov.uk/peoplepopulationandcommunity/culturalidentity/ethnicity/bulletins/ethnicgroupenglandandwales/census2021.

